# Right hepatectomy due to portal vein thrombosis in vasculobiliary injury following laparoscopic cholecystectomy: a case report

**DOI:** 10.1186/1752-1947-8-412

**Published:** 2014-12-07

**Authors:** Stipislav Jadrijevic, Davorin Sef, Branislav Kocman, Anna Mrzljak, Hrvoje Matasic, Dinko Skegro

**Affiliations:** 3 Department of Surgery, Division of Transplantation Surgery, University Hospital Merkur, Zagreb, Croatia; 4 Department of Medicine, University Hospital Merkur, Zagreb, Croatia; 5 Department of Anesthesiology and Critical Care, University Hospital Merkur, Zagreb, Croatia; 6 School of Medicine, University of Zagreb, Zagreb, Croatia

**Keywords:** Bile duct injury, Hepatectomy, Hepatic artery injury, Laparoscopic cholecystectomy, Portal vein thrombosis, Vasculobiliary injury

## Abstract

**Introduction:**

Vasculobiliary injury composed of bile duct, portal vein and hepatic artery injury is a rare, but the most severe, complication after cholecystectomy that may require hepatectomy or even urgent liver transplantation.

**Case presentation:**

We present a case of a 36-year-old Caucasian woman with a biliary sepsis and a large right liver lobe abscess due to an extreme vasculobiliary injury after laparoscopic cholecystectomy. Bismuth type IV biliary duct injury, portal vein thrombosis and injury of right hepatic artery were identified, resulting in life-threatening septic episodes. Right hepatectomy with Roux-en-Y hepaticojejunostomy and reconstruction of her portal vein with a vein allograft were performed. She fully recovered and remained well during 3 years of follow-up.

**Conclusions:**

Although rare, the impact of vasculobiliary injuries after cholecystectomy highlights the need for constant alertness and prompt management in order to minimize the risk of the routine operative procedure. Hepatectomy with biliary and vascular reconstruction should be considered early in the management of vasculobiliary injury to avoid the development of life-threatening consequences.

## Introduction

Laparoscopic cholecystectomy (LC) is the procedure of choice for symptomatic cholelithiasis, but is associated with a higher incidence of iatrogenic bile duct injuries than the open procedure [1, 2]. Vasculobiliary injury (VBI), defined as bile duct, hepatic artery and/or portal vein injury, has been recognized as one of the most severe complications after cholecystectomy that may result in various degrees of hepatic ischemia with subsequent liver necrosis, abscess formation, acute liver failure or secondary biliary cirrhosis. [3–5]. Biliary tree anomalies present in up to 25% of patients [6], and may lead to anatomical misidentification and technical problems that contribute to the development of these injuries. The management of VBI depends considerably on development of biliary ischemia and hepatic infarction, necessitating in some cases hepatectomy or even urgent liver transplantation [7]. However, the best treatment strategy and timing of surgical repair when there is a VBI is still controversial.

Here we present a management of VBI after LC. The presented case emphasizes the need for prompt and adequate management of patients with VBI in order to avoid life-threatening complications.

## Case presentation

**Figure 1:**
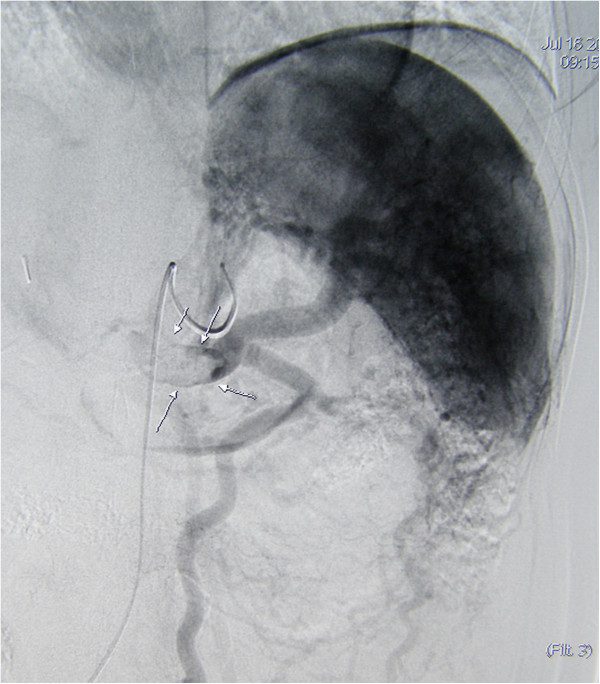
**Angiography shows occlusion and thrombosis in the portal vein down to the splenomesenteric confluence (arrows).**

**Figure 2:**
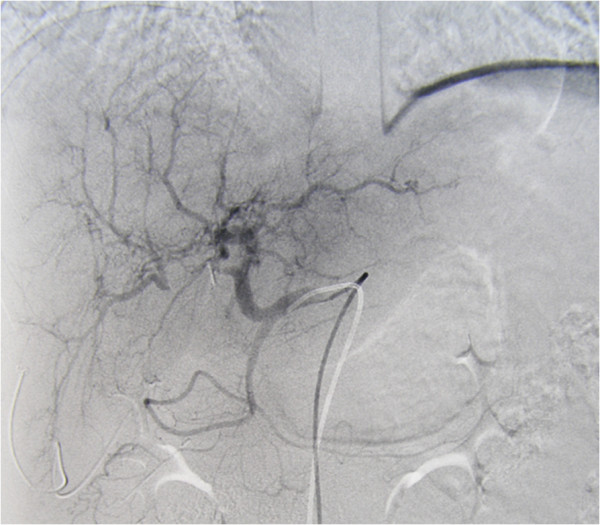
**Angiography after laparoscopic cholecystectomy showing occlusion of right hepatic artery.**

**Figure 3:**
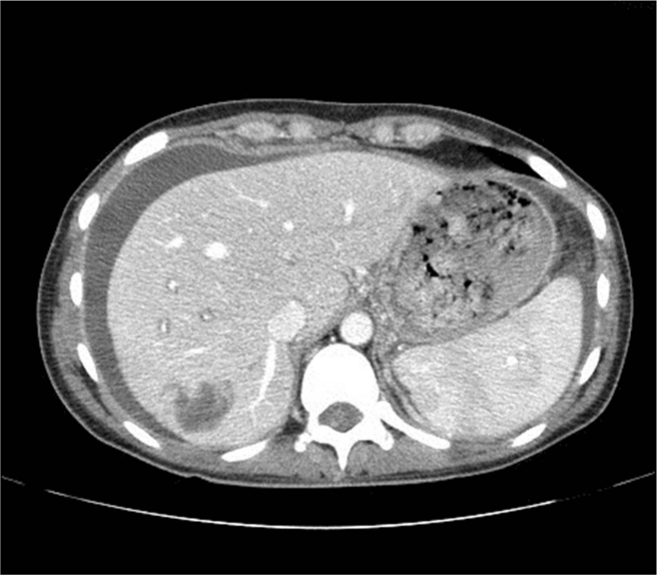
**Abdominal computed tomography image showing large right liver lobe abscess accompanied by perihepatic and interintestinal biloma.**

A previously healthy 36-year-old Caucasian woman underwent early LC for an acute cholecystitis in an affiliated hospital. Due to uncontrolled bleeding, LC was converted to an open procedure through a standard right subcostal laparotomy. Hemostasis was achieved using sutures and clips, and common bile duct injury was repaired using a T-tube. Postoperatively she became febrile and complained of right upper abdominal pain; a laboratory examination revealed cholestatic profile of liver tests (total bilirubin 99.3μmol/L, alkaline phosphatase 207IU/L, gamma-glutamyl transferase 209IU/L) and increased inflammatory markers (C-reactive protein 129.4mg/L). Secondary cholangiography showed a Bismuth type IV common bile duct transection with biliary leak along the T-tube. Blood cultures tested positive for methicillin-resistant *Staphylococcus aureus* and *Acinetobacter baumannii*, and parenteral therapy with vancomycin and colistin was initiated. Portal vein thrombosis (Figure 1) and right hepatic artery injury (Figure 2) were identified by hepatic angiography. Four weeks after the LC, the patient developed systemic inflammatory response syndrome (SIRS) and was transferred to our hospital. Abdominal computed tomography confirmed the previous findings, as well as a large right liver lobe abscess accompanied by perihepatic and interintestinal biloma (Figure 3).

**Figure 4:**
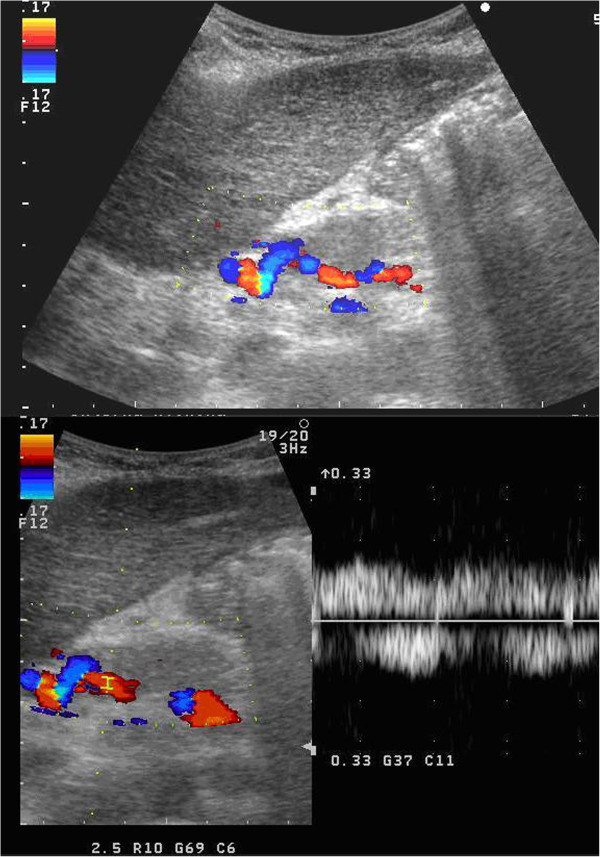
**Abdominal Doppler ultrasound 2 years after right hepatectomy with reconstruction of portal vein showing normal liver parenchyma and good graft patency.**

The further surgical management included right subcostal laparotomy revealing a diffuse peritonitis with severe inflammatory reaction of her hepatic hilum, a large biloma and right hepatic duct and right hepatic artery suture ligatures as well as portal vein suture ligatures with thrombosis above the splenomesenteric confluence. Based on these findings, right hepatectomy with a left Roux-en-Y hepaticojejunostomy was performed. Due to the distal portal vein thrombosis and portal vein suture ligatures (above the splenomesenteric confluence) reconstruction with cadaveric iliac vein allograft was performed. A 6-day-old vein allograft of identical blood group and anatomical match was used. The postoperative period was further complicated with the biliary sepsis, bilateral pleural effusions and pneumonia that subsequently resolved. She was discharged from the hospital 60 days after the hepatectomy, fully recovered. After 2 years of follow-up, her liver function tests are unremarkable with patent allograft as demonstrated by Doppler ultrasound (Figure 4).

## Discussion

A VBI as an injury of a bile duct and adjacent vascular structures is mainly caused by operative trauma during cholecystectomy. Injuries to the bile duct and liver vessels may occur due to mistakes in dissection, so their identification and careful dissection around the neck of the gallbladder is the best way to avoid them. Common bile duct or aberrant right hepatic ducts are most often misidentified as the cystic duct. The most dangerous biliary anomaly is the cystic duct that runs along the side of a low-lying aberrant right segmental duct. Most commonly this is the right posterior hepatic duct, occurring in 2.5 to 8% of patients, which drains liver segments 6 and 7 [3]. Therefore, performing meticulous dissection around the gallbladder neck and cystic duct before clip placement enables visualization of any variant anatomy. In up to 92% of cases, biliary injury is accompanied by injury of the right hepatic artery [8]. In rare cases, the portal vein alone or in combination with other arteries is involved. These data are variable in some reports due to the fact that angiography was mostly performed selectively. Therefore, routine hepatic arteriography is recommended in all patients with biliary duct injury after cholecystectomy if early repair is considered [8]. Further on, since hepatic parenchyma depends on portal circulation in cases of arterial injury, examination of the portal blood flow is necessary in any case of VBI [9].

So far, the data regarding specific type of VBI composed of biliary duct, portal vein and hepatic artery are scarce, especially in terms of management and outcome [8–12]. Portal vein resistance to injury or under-reporting of such cases due to rapid clinical deterioration and death may be some of the reasons [8]. Portal vein thrombosis in VBI is in the majority of cases accompanied by injury of a major hepatic artery, predominantly the right hepatic artery [8]. This type of VBI, compromising dual hepatic supply, results in more rapid and severe hepatic ischemia compared to isolated arterial injury. The treatment of this potentially lethal complication might require liver resection or even liver transplantation [8–11, 13, 14]. Unrecognized VBI in patients who remain asymptomatic can lead to development of biliary strictures, cholangitis and liver atrophy [14].

Current data demonstrate that rapid liver necrosis in almost half of patients with VBI with dual vascular injury resulted in right hepatectomy within the first 2 weeks after the injury with a mortality rate of 50% [8]. In our patient, uncontrolled clipping and suturing during the cholecystectomy resulted in right biliary duct and hepatic artery injury with subsequent portal vein thrombosis. Right hepatectomy was performed 4 weeks after the development of VBI, however, in a critically ill patient SIRS developed due to biliary sepsis and postponed treatment of the liver abscess. In our case, portal vein resection and reconstruction using a vein graft had to be performed due to partial iatrogenic injury and thrombosis of the portal vein. The prompt management and early referral to a tertiary center is therefore of paramount importance since the consequences of rapid hepatic ischemia in this type of injury may be devastating.

Liver transplantation is still a controversial option for patients with complex VBI, mainly because of concomitant septic complications and low survival rates, although recently it has been reported in the treatment of long-term complications such as secondary biliary sclerosis [7].

## Conclusions

Considering the reported rarity on the one hand and its devastating consequences on the other hand, it is important to compile the evidence and experience regarding portal vein thrombosis in VBI, in order to establish proper management strategies and improve patients’ outcomes. Therefore, the present case contributes to the emerging literature about this issue emphasizing the need for the prompt management and early referral to a tertiary center where hepatic resection with biliary and vascular reconstruction can be safely performed.

## Consent

Written informed consent was obtained from the patient for publication of this case report and accompanying images. A copy of the written consent is available for review by the Editor-in-Chief of this journal.

## Abbreviations

LC: Laparoscopic cholecystectomy
SIRS: Systemic inflammatory response syndrome
VBI: Vasculobiliary injury.
